# Citizens’ perspective on ‘the right care in the right place’

**DOI:** 10.1093/eurpub/ckac129.460

**Published:** 2022-10-25

**Authors:** L Damen, JD De Jong, L Van Tuyl, JC Korevaar

**Affiliations:** 1 NIVEL, Utrecht, Netherlands; 2 Maastricht University, Maastricht, Netherlands

## Abstract

**Background:**

Healthcare systems around the globe are facing challenges, from increasing demand and costs to a diminishing health workforce. Without change, healthcare will become unsustainable. In the Netherlands, the government aims to organize sustainable healthcare by among others the policy ‘the right care in the right place’. An important part of this policy is relocating healthcare, for instance, from the hospital to the general practitioner (GP) or from the GP to other healthcare providers or to citizens themselves. Relocation of care is expected to reduce costs and manpower shortages. There is, however, little known about how citizens think about this topic, although they are an important stakeholder. This research aims to investigate citizens’ perspectives on the right place for care.

**Methods:**

A questionnaire was sent in December 2021 to 1.500 members of Nivel's Dutch Healthcare Consumer Panel, including 4 questions about the right care in the right place. The response was N = 796 (53%). In addition, two citizen platforms were organized in March 2022, to discuss the right care in the right place. A total of 23 citizens participated.

**Results:**

First results indicate that most citizens related expertise and accessibility to the right care in the right place. Participants said that non-complex care could be relocated from the hospital to the GP if the GP has the right expertise. Expertise was mentioned as the most important aspect of care delivery. According to participants, care could be shifted from the GP to other primary care professionals, to social services or to self-sustainability, given that citizens know who to visit or what to do with health problems/worries. More results will be available by November.

**Conclusions:**

Relocating care could be a possible solution to keep healthcare sustainable in the future in terms of costs and manpower. Among citizens, there seems to be support when certain conditions are met.

**Key messages:**

• Citizens support the substitution of non-complex care from hospitals.

• Citizens are willing to improve self-sustainability when they have the tools.

